# Using optical emission spectroscopy in atmospheric conditions to track the inflight reduction of plasma sprayed TiO_2−x_ feedstock for thermoelectric applications

**DOI:** 10.1038/s41598-023-50592-5

**Published:** 2024-01-04

**Authors:** Georg Mauer, Edward J. Gildersleeve V

**Affiliations:** Institute of Energy and Climate Research, IEK-1: Materials Synthesis and Processing, Forschungszentrum Jülich GmbH, 52425 Jülich, Germany

**Keywords:** Engineering, Energy infrastructure, Thermoelectrics, Materials science, Optical spectroscopy

## Abstract

Thermal spray deposition (specifically Atmospheric Plasma Spraying, APS) is a well-established surface coating technology with a broad scope of applications (i.e., insulative coatings, tribological coatings, anti-corrosion coatings, etc.). In addition, there is a constant drive to introduce the APS process into new and emerging fields. One such niche application for APS would be sub-stoichiometric TiO_2−x_ coatings with enhanced thermoelectric performance (compared to the bulk material). The APS process in this context has a unique ability—given the use of hydrogen as a plasma gas—to reduce TiO_2−x_ material during processing. However, to this point, there is neither a reliable nor self-consistent method to assess (nor control by parametric optimization) the inflight reduction of molten oxide particles during processing. This study shows that using Optical Emission Spectroscopy (OES), it can be possible—even in atmospheric conditions—to identify characteristic emission peaks associated with the inflight reduction of TiO_2_ during APS. Using this OES data, the input spray processing parameters and their influence on coating microstructure and the degree of inflight reduction of the material will be shown. Results suggest under equilibrium conditions only a minimal amount of hydrogen gas is needed in the plasma to fulfill the TiO_2_ reduction.

## Introduction

Thermal spray deposition is a longstanding process which allows one to fabricate functional surface coatings atop a wide array of substrates (metallic, ceramic, polymer, etc.)^1,2^. Popular applications of the technology include, but are not limited to, wear-resistant coatings (i.e., for the paper rolling industry, aircraft landing gear, etc.), anti-corrosion coatings (i.e., for naval environments, radioactive waste environments, etc.), and high-temperature resistant coatings (i.e., for gas turbine engines, spacecraft re-entry, hypersonics, etc.)^2–4^. In addition to these well-established applications, researchers in academia and industry alike continue to search for opportunities to expand the technology space further into more specialized applications.

The concept of ‘thermal spray deposition’ is an all-encompassing term which covers a broad range of manufacturing processes. In general, ‘thermal spraying’ means to take a feedstock material, i.e., in solid form, such as powder granules, and—either by melting and rapid resolidification or by successive plastic deformation/peening—continually stack ‘building blocks’ called splats of material atop one another to build a surface coating. Thermally-sprayed coatings are usually meso-scale, on the order of microns to millimeters in thickness. These thermal spray processes are typically further classified according to their range of achievable particle temperatures and velocities^1,2,5^.

In this work, the Atmospheric Plasma Spraying (APS) process was utilized. In the APS process, a plasma torch is used to deposit coatings—wherein an electric arc is generated between the anode and cathode within the torch. Concurrently, a mixture of gases (typically argon, hydrogen, helium, nitrogen) are projected through the torch body that ionize once exposed to the electric arc and form a plasma outside the anode orifice. Feedstock material is then injected into this plasma source to achieve melting, and the gas momentum projects the molten droplets toward a surrogate substrate—where rapid solidification and deposition of the coating occurs^1,2,5^. The APS process is one of the most versatile thermal spray processes, as the predominant mechanism of coating formation (melting and rapid solidification) allows for a highly flexible range of materials to be sprayed.

Beyond the aforementioned popular industrial applications of thermal spraying, one example of a more specialized use of the technology is depositing sensors, electronics, and thermoelectric materials using the APS process^3,6–10^. For these applications, the coating layer itself acts as the functional body (i.e., either sensing gaseous species, generating electricity, or acting as resistors). Presently, the use of plasma spray deposition to form meso-scale functional sensor materials has seen commercial success [Mesoscribe Corporation, CVD Equipment Corporation, Central Islip USA]. Nonetheless, there is typically a narrow range of allowable material chemistries and phases which are acceptable for such desired properties. For example, in the thermoelectric community (where the goal is to convert waste heat into usable energy), one popular material of interest is sub-stoichiometric titanium oxide (TiO_2−x_). Due to its oxygen substoichiometry combined with being an oxide material, TiO_2−x_ can have enhanced electrical conductivities and reduced thermal conductivities that contribute to enhanced thermoelectric performance^11^. Aside from thermoelectrics, sub-stoichiometric TiO_2−x_ also has unique applications in wear and tribology, due to the formation of Magnéli phases that are intrinsically more lubricative than the other TiO_2_ polymorphs^12^.

**Figure 1:**
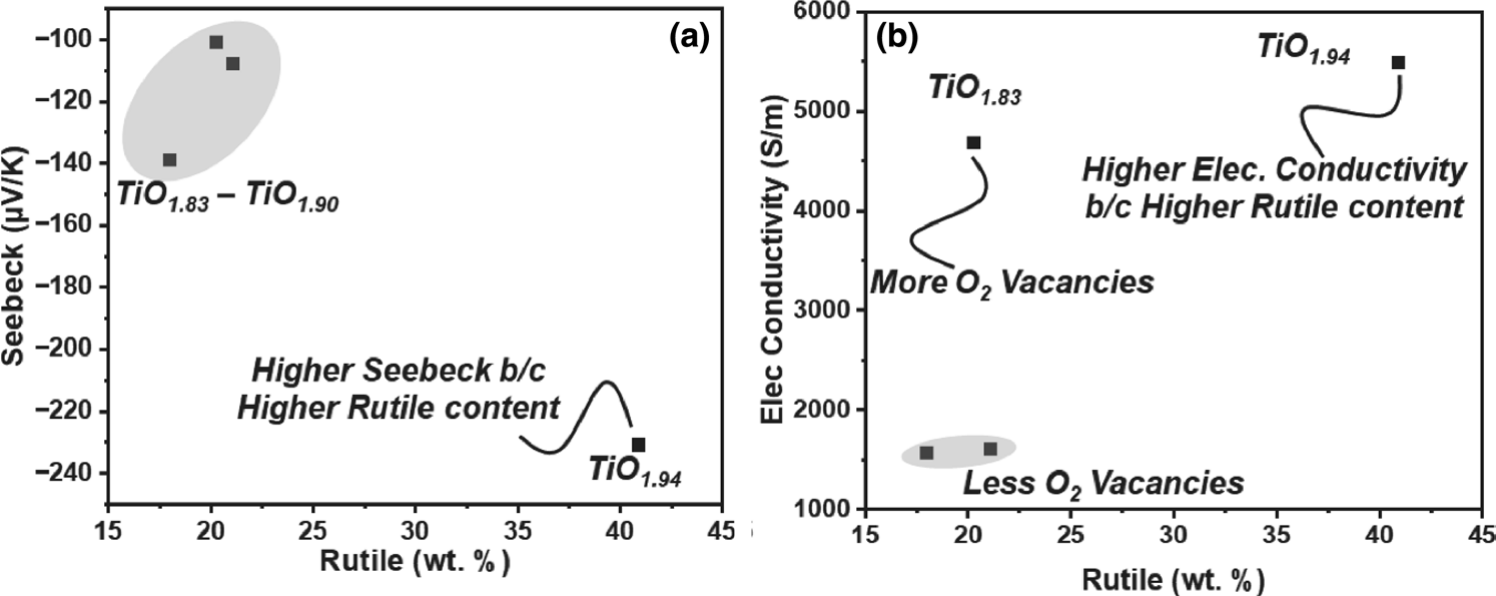
(**a**) Seebeck Coefficient of plasma-sprayed TiO_2−x_ coatings as a function of Rutile phase content (as determined by Rietveld Analysis of X-Ray Diffraction data). (**b**) Electrical conductivity of plasma-sprayed TiO_2−x_ coatings as a function of Rutile phase content. The oxygen content of the TiO_2−x_ coatings is overlaid in the plots. Data extracted from Lee et al.^13^.

The concept of TiO_2−x_ is an example of such applications where sub-stoichiometric oxide materials have niche functional properties which are typically unachievable by conventional materials processing routes, yet are possible via thermal spray deposition. Considering the use of conventional argon–hydrogen (Ar–H_2_) gas mixtures in the APS process, a material such as TiO_2_ should conceivably reduce during spraying to TiO_2−x_. However, the extent to which this in-situ, inflight reduction can occur—and how the processing parameters (i.e., gas flow rates, gas mixtures, etc.) directly contribute to how much reduction occurs is, to this point, unclear. Lee et al. has shown in several works that by modulating the hydrogen content in the plasma gas mixture, the TiO_2−x_ substoichiometry can be somewhat controlled^10,13,14^. Furthermore, these studies have also examined the thermoelectric properties of plasma-sprayed substoichiometric TiO_2−x_ coatings; and found that indeed the more heavily reduced TiO_2−x_ coatings exhibit higher electrical conductivities^10,13,14^. This can be seen in Fig. 1b. Figure 1 also shows the complex interplay between TiO_2−x_ substoichiometry and rutile phase retention. While more oxygen substoichiometry is desired from an electrical conductivity point of view, the formation of Magnéli phases is disadvantageous to the coating’s Seebeck Coefficient—which can negatively impact the coating’s Figure of Merit $ZT$^15,16^.

Clearly, then, there is a narrow thermal spray processing window wherein desirable oxygen reduction and concurrent rutile phase retention exists. However, to date, there is no cohesive methodology that for users to identify the in-situ reduction phenomena. One study has been recently published which utilizes Optical Emission Spectroscopy (OES) to try and observe the characteristic emission lines of Ti and Ti–O during spray processing^17^. However, this study does not focus on the APS process specifically, nor does it consider the influence of processing parameters such as hydrogen content in the plasma gas mixture on the final results.

This study strives to examine deliberate and systematic changes in the APS processing conditions for a given TiO_2_ material and simultaneously ascertain the relationships in processing parameters and inflight reduction using OES. Specifically, the processing parameters utilized by Lee et al. in past works are reproduced here in order to identify in-situ decomposition mechanisms for thermal spray processes which have had concurrent thermoelectric property measurements^14^. In contrast to low pressure conditions, the application of OES at atmospheric pressure is principally challenging since the continuum background radiation is larger, and significant peak broadening occurs^18^. In this study, it was found that a particular set of titanium emission lines is suitable to derive plasma temperatures, even in such disadvantageous conditions. Using these data, the overall concentration of evaporated species in the plasma jet originating from the feedstock was also determined. Moreover, the electron density and characteristic plasma parameters were calculated to assess the plasma state. Coatings were also fabricated to study the process parameters’ influence on the formation dynamics in terms of phase and microstructure.

## Results and discussion

Using the methodologies and calculations laid out in the “Methods” section, it was possible to take the experimental OES data generated for each of the cases in Table 1 and assess both the plasma temperatures and the semi-quantitative emission of species from the vaporization of the TiO_2_ feedstock at multiple torch standoff distances.

**Table 1:** Investigated process parameters for spray experiments.

Case	Current (A)	Ar (slpm)	H_2_ (slpm)	*P*_in_ (kW)	*P*_net_ (kW)	*η* = *P*_net_⋅*P*_in_^−1^	*h* (MJ kg^−1^)	C/G (slpm)
1	550	47.5	0	18.4	9.1	50%	6.4	4
2	550	47.5	6	34.0	19.7	58%	13.9	3.5
3	550	65	6	35.3	22.4	64%	11.5	6
4	550	65	11	39.1	24.9	64%	12.8	7

Slpm, standard liters per minute; *P*_in_, electrical input power; *P*_net_, torch net power; *η*, torch efficiency; *h*, mass specific enthalpy; C/G, carrier gas.

### Coating and spray process characterization

**Figure 2:**
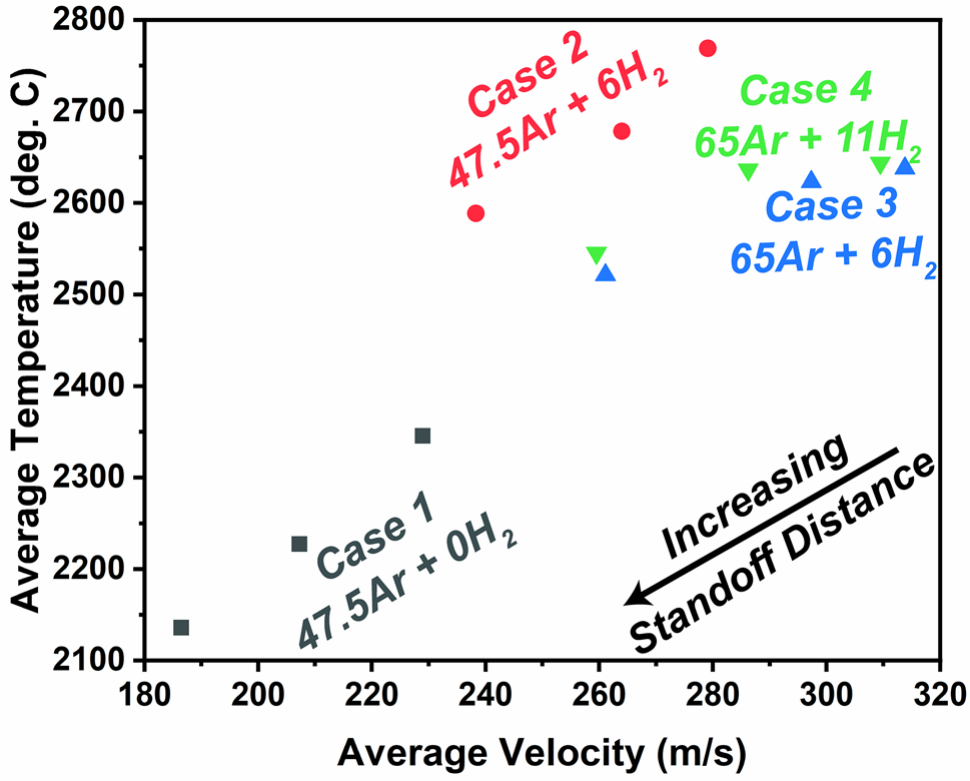
Average particle temperatures and velocities for the four cases in Table 1, measured at the three standoff distances (60, 80, 100 mm) of interest in this study.

For all suitable OES conditions, the particle states (temperature and velocity) were measured using the methods mentioned in the experimental section. Figure 2 shows the average particle temperatures and velocities for the conditions of interest, which are in agreement with past values measured by Lee et al., suggesting the particles here have undergone similar thermal excursions as they have in their past work^14^. Clearly from the Figure, the argon-only case 1 (Table 1) yielded substantially lower particle temperatures than any of the Ar–H_2_ conditions. This is an expected result, as the addition of hydrogen tends to increase the flame enthalpy as well as plasma intensity^19^. In cases 3 and 4, where higher argon flow rates were utilized, particles were seen to have nominally higher velocities. These increased velocities, which implies reduced particle residence time within the flame, inevitably contributed to the lower particle temperatures—as compared to case 2.

**Figure 3:**
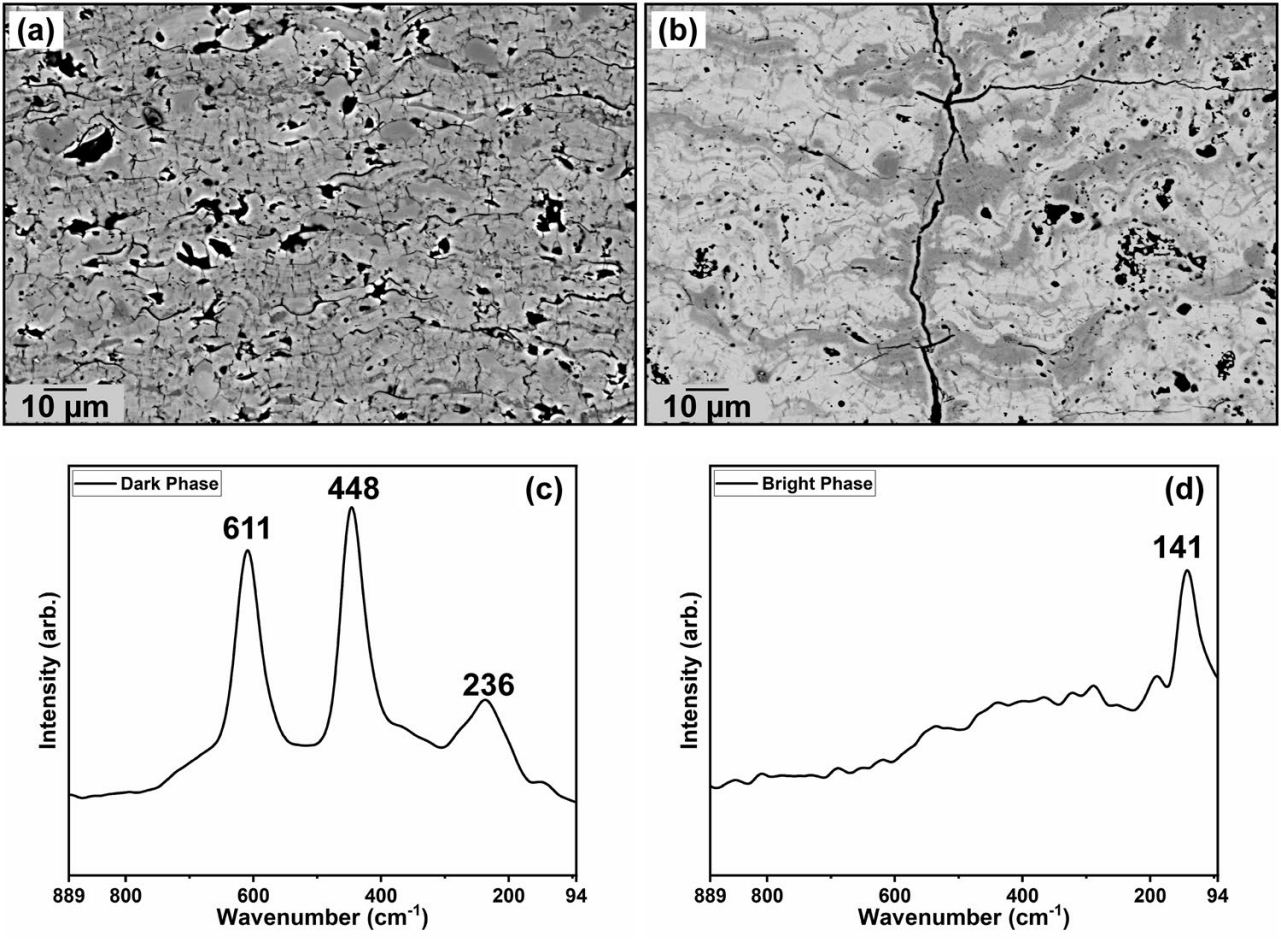
(**a**,**b**) Cross-sectional backscatter electron microstructures of APS TiO_2_ coatings sprayed at 60 mm standoff distance with the fine feedstock, using conditions from case 1 and case 2, respectively. (**c**) and (**d**) show Raman spectra from the coating (**b**) from within the dark and bright phases, respectively.

Coating backscatter electron cross-section micrographs can be seen for case 1 and case 2 in Fig. 3—both sprayed at a 60 mm standoff distance. From the Figure, there is a clear difference in the overall microstructure of the coatings. In the argon-only case 1, the TiO_2_ coating exhibits characteristics of a traditional thermally sprayed coating microstructure. That is, there is a heterogenous distribution of pores and microcracks present in the coating. However, interestingly, in the case 2 (Fig. 3b), there is a distinct heterogeneity in backscatter contrast in the image. EDS analysis confirmed that the chemical differences between these two phases (in Ti concentration) was less than 2 wt.%, which is not enough to account for the observed contrast differences. Nonetheless, it can be said from this data that the bright phase is relatively more ‘reduced’ than the dark phase.

More importantly, Raman Spectroscopy revealed that this heterogeneous backscatter contrast is more likely a consequence of the dispersion of different TiO_x_ phases within the coating. Figure 3c and d show that the darker phase in the coating from Fig. 3b has a characteristic rutile Raman spectrum, whereas the bright phase is much more structurally disordered. From the bright phase spectrum, there is possibly one characteristic peak present; this peak has been ambiguously defined in the literature to be indicative of either anatase TiO_2_ or Magnéli Ti_n_O_2n−1_ phases^20–22^. The microstructural anomalies shown here were investigated in more detail in a concurrent study, and are shown here in this paper primarily as an illustrative example of how plasma spray processing can influence the structure and properties of TiO_2−x_ coatings^23^. The following discussion will examine the in-situ OES findings and how they may provide insight toward what has been observed cross-sectionally and in traditional thermal spray process diagnostics such as particle temperature and velocity measurements.

### Calculated plasma temperatures

**Figure 4:**
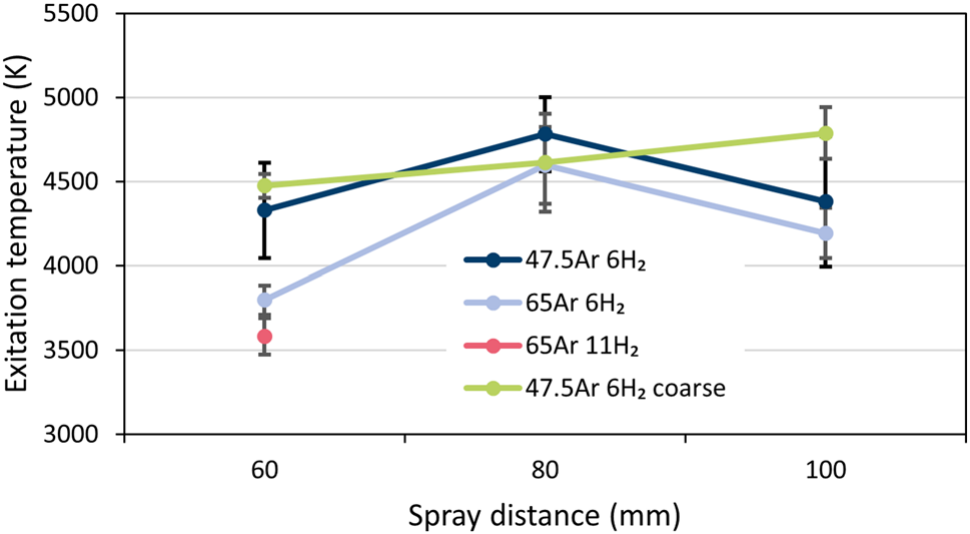
Excitation temperatures determined by OES for the four experimental cases across three spray distances; the error bars represent the uncertainty estimated on the basis of the standard error of the slope of the regression line in the Boltzmann plots.

As mentioned in the “Methods” section, the standoff distance effect could only be studied based on the Ti I-lines at distances between 60 and 100 mm. In this range, the variation in the excitation temperature with the spray distance was fairly moderate, see Fig. 4. Interestingly, the highest plasma temperatures were found for the plasma gas mixture of 47.5Ar + 6H_2_ (case 2), followed by 65Ar + 6H_2_ (case 3) and 65Ar + 11H_2_ (case 4). This finding is important as it is somewhat counter-intuitive to anecdotal APS processing knowledge—wherien generally operators will use higher hydrogen flow rates to achieve more particle melting (effectively assuming the plasma flame temperature increases).

The reasons for this surprising deviation from anecdotal knowledge are assumed to be as follows. Increasing the Ar flow rate (with constant 6 slpm hydrogen (case 2 → case 3) from 47.5 to 65 slpm effectively lowered the mass specific plasma enthalpy from 13.9 to 11.5 MJ kg^−1^ (see Table 1). Meanwhile, increasing the H_2_ flow rate (case 3 → case 4) from 6 to 11 slpm with a constant 65 slpm Ar flow rate increased the inherent thermal conductivity of the plasma gas mixture, while only slightly changing the mass specific plasma enthalpy from 11.5 to 12.8 MJ kg^−1^. The net change in enthalpy is reduced with increasing hydrogen flow rates as compared to increasing argon flow rates due to constraints imposed by the contraction of the arc. One can also note the torch efficiency *η* = *P*_net_/*P*_in_ was stable at 64% for both cases 3 and 4 (see Table 1). Thus, the increased plasma thermal conductivity must have induced thermal losses to the ambient atmosphere in the case 4. The practical outcome of these marginal changes induced by increased hydrogen flow rate in case 4 can also be seen in the statistically insignificant differences in particle state data between case 3 and case 4 (see Fig. 2). It was also interesting to find that when feeding the coarser powder, the calcualted plasma temperatures were quite similar to those for the finer powder. This implies for these given mass feed rate conditions (5 g min^−1^), there seems to be no effect of ‘particle loading’ that might thermally-quench the plasma^24^.

### Estimation of the plasma state

Figure 4 shows that the plasma temperatures fall within similar ranges over the torch standoff distances studied here, which is a somewhat surprising result. Typically, as the torch standoff distance increases, the available thermal energy should decrease. To further investigate the trends found in this work, a more detailed study on the plasma state—and its potential influence on the plasma temperatures—was carried out for the given torch conditions using the equations laid out in the “Methods” section.

The ionization degree in thermal spray plasma jets is generally low (< 1–3%) due to their relatively low temperatures^25^. Hence, the electron densities *n*_e_ calculated acc. to Eq. (6) for all investigated experimental cases and spray distances as 5⋅10^16^ m^−3^ to 1⋅10^19^ m^−3^ were only moderately large. These are typical values for “medium density” plasmas^26^. The calculated *n*_e_ results show the same ranking as the plasma temperatures given in Fig. 4.

The condition for a continuous plasma is fulfilled since the plasma parameters Λ (calculated by Eq. 9) for all experimental cases and spray distances were determined to be between 130 and 1323, which is significantly larger than unity. These Λ values showed an inverse ranking as compared to the plasma temperatures.

The average distances $\overline{x }$ between ions and electrons calculated by Eq. (11) were between 0.3 and 1.7 µm which are quite large, since this is 115 to 542 times the Landau length *λ*_L_, calculated acc. to Eq. (10) to be between 2.3 and 3.1 nm. Similar to the plasma parameter Λ, the factors $\overline{x }/{\lambda }_{L}$ trend inversely as compared to the plasma temperatures in the investigated cases of this work. The relation $\overline{x }>{\lambda }_{L}$ implies that recombinations of ions and electrons are prevented.

On the other hand, the Debye lengths were calculated acc. to Eq. (8) to be between 1.5 and 18.5 µm. Since the average distances $\overline{x }$ between ions and electrons were less than the corresponding Debye lengths *λ*_D_,_,_ electrostatic interactions of the plasma particles and hence collective interaction phenomena occur as they are typical for a plasma.

**Figure 5:**
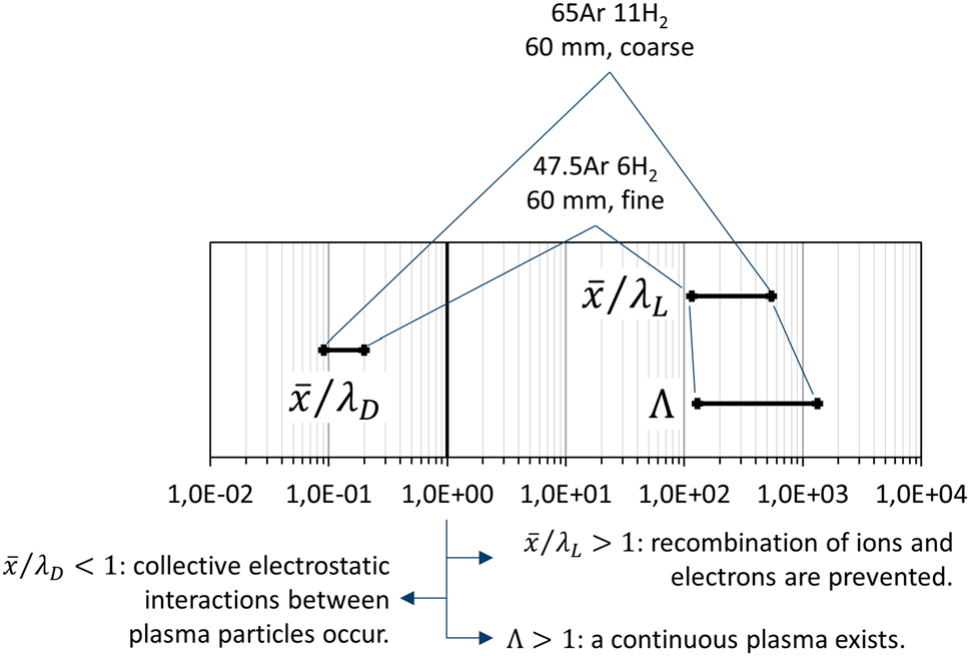
Ranges of the calculated dimensionless ratios of characteristic lengths and the plasma parameter for all investigated experimental cases and spray distances. Symbols are explained in the text; the critical value of unity is highlighted.

The calculated ranges of the characteristic lengths and the plasma parameter Λ are summarized for all investigated experimental cases and spray distances in Fig. 5. The extrema were found for case 4 (65Ar 11 H_2_, 60 mm, coarse powder) and for case 2 (47.5Ar 6H_2_, 60 mm, fine powder), respectively. The data points of all other investigated cases lie in between. As shown, the conditions are such that a true plasma exists and recombinations of ions and electrons hardly occur. Therefore, ionization energies are only released to a limited extent and the plasma temperatures should remain stable over long standoff distances, as seen in Fig. 4. Obviously, the condition of such a ‘hot and diffuse’ plasma essentially persists throughout the plasma jet.

### Identified plasma species

First, OES measurements were conducted without any powder injection into the plasma. In the pure argon plasma (case 1, see Table 1) emission lines of neutral and single ionized argon Ar I and Ar II, respectively, could be identified. Furthermore, neutral atomic and molecular nitrogen N I and N_2_, respectively, were also found, obviously originating from ambient air entrained into the jet. No oxygen lines were found, nor were there emission lines of vaporized tungsten or copper (which would originate from degradation of the torch electrodes). Adding hydrogen (cases 2, 3, and 4) results in the formation of additional atomic hydrogen lines (i.e., the Balmer series). However, no lines of the hydroxyl radical OH or water vapor H_2_O were identified. The detection of molecular hydrogen H_2_ using these conditions and techniques is ambiguous since 8300 potential emission lines exist.

**Figure 6:**
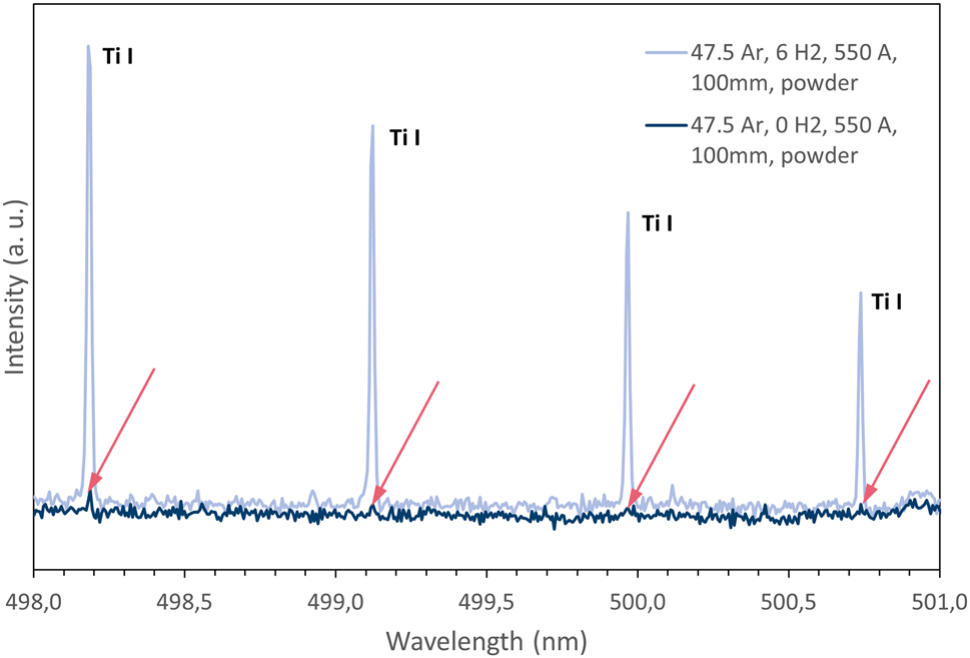
Detail of the emission spectra for the experimental cases 1 (Ar only) and 2 (6 slpm H_2_ addition) with TiO_2_ feedstock injection showing the four most powerful Ti I lines measured at 100 mm spray distance; the arrows indicate the minute Ti I emission lines for case 1.

When operating the plasma torch with the TiO_2_ feedstock powder, emission lines for neutral Ti I atoms and single ionized Ti II ions appear, see Fig. 6. For the Ar-only plasma (case 1) these Ti emission lines are present, but clearly are too miniscule to be evaluated properly using the techniques described in the “Methods” section. Obviously from this result, one can conclude TiO_2_ hardly decomposes and evaporates in this argon-only plasma case. By contrast, there are considerable amounts of Ti vapor present once hydrogen is introduced in the plasma. This is consistent with expectations that hydrogen should contribute to inflight reduction of TiO_2_ feedstock during APS processing. Furthermore, the works by Lee et al. also show that the APS process—when iterating between Ar-only and Ar–H_2_ plasmas, yields a distinct change in the amount of TiO_2_ reduction^10,13,14^.

**Figure 7:**
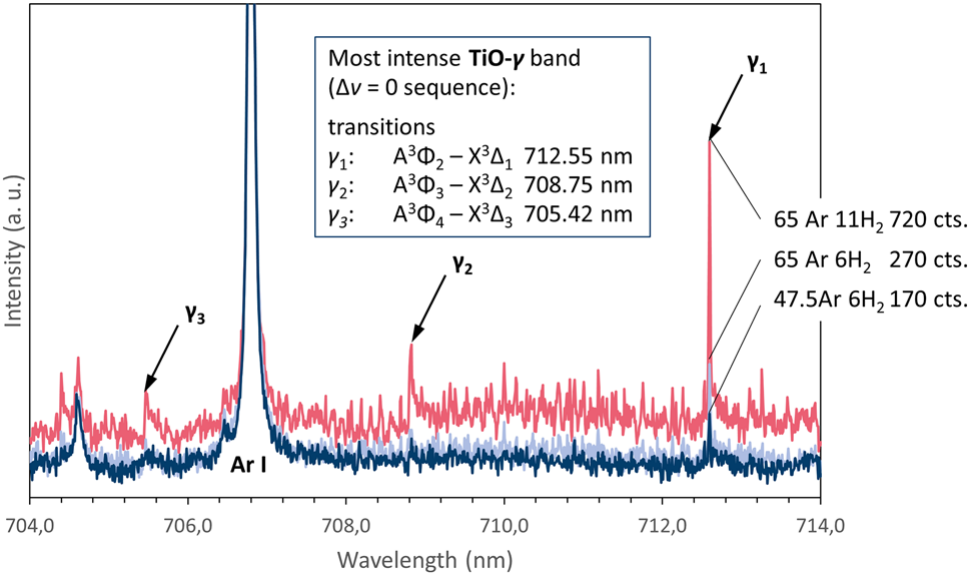
Detail of the emission spectrum for the Ar–H_2_ experimental cases 2, 3, and 4 measured at 100 mm spray distance with the most intense TiO-*γ* band (Δ*ν* = 0 sequence) with three transitions at 705.42 nm, 708.75 nm, and 712.55 nm indicated by arrows.

Furthermore, the presence of the TiO radical could be verified in these hydrogen-containing-plasma datasets, likely existing as a decomposition byproduct of the TiO_2_ feedstock. Figure 7 shows the most intense TiO-*γ* band (Δ*ν* = 0 sequence) with three electronic transitions at 705.42 nm, 708.75 nm, and 712.55 nm. The peak heights are seen to increase with increasing plasma power. For more details on the transition bands of TiO, see^27^.

### Chemical equilibria of TiO_2_ in Ar-based plasmas

To explain the temperature-dependent melting, vaporization, and dissociation behavior of TiO_2_ into the observed sub-species, chemical equilibria were calculated by minimizing Gibb’s energy for all of the experimental cases using the software CEA^28,29^. While the results for case 1 stand alone as unique, the Ar–H_2_ cases 2, 3, and 4 showed comparable equilibria; hence, only results for case 2 are shown here as an example.

**Figure 8:**
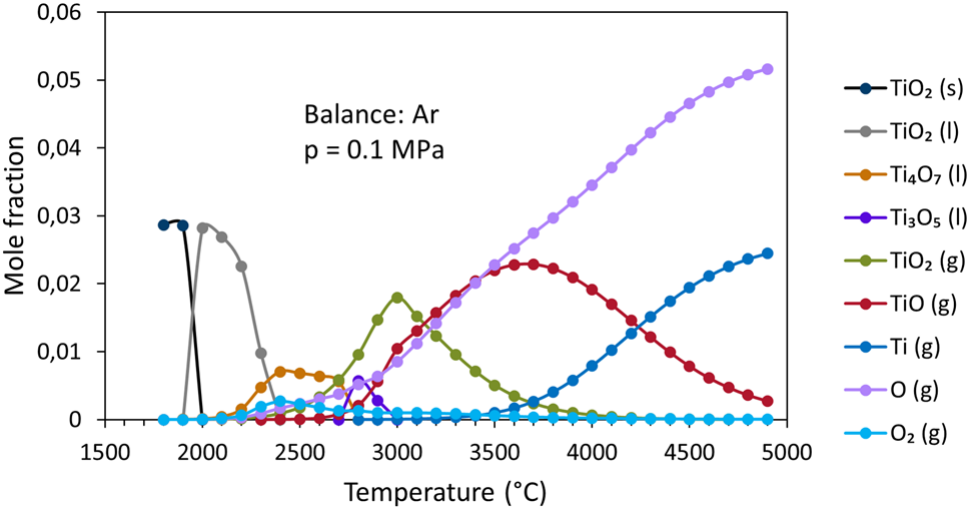
Temperature-dependent melting, vaporization, and dissociation of TiO_2_ in Ar-only plasmas (case 1) at chemical equilibrium.

Figure 8 shows the results of chemical equilibria for case 1 (argon-only plasma). Rutile TiO_2_ melts at 1912 °C,^30^ and subsequently dissociates to liquid Magnéli phase compositions Ti_4_O_7_ and Ti_3_O_5_. The first evaporation product is initially TiO_2_, but dissociates at higher temperatures to TiO + O and finally to Ti + O. The onset of Ti vapor formation was determined to occur only at approx. 3200 °C. It is possible to derive a basic understanding of the chemical composition of the molten TiO_2−x_ material in the plasma by examining the maximum particle temperatures achieved for these argon-only conditions, which was only ~ 2400 °C (Fig. 2). From this cross-examination between particle state and chemical equilibria, the miniscule Ti emission lines in Fig. 6 can be more clearly explained. If the particles never superseded the temperatures required to dissociate TiO_2_ to sub-stoichiometric liquid phases, then it stands to reason the microstructural outcome should be phase-homogeneous, as it is in Fig. 3a. Furthermore, the particle state data from Fig. 2 also shows that none of the Ar-only spraying conditions’ average particle temperatures came close to the > 3200 °C temperatures required to dissociate TiO_2_ (gas) into TiO + O and Ti + O.

**Figure 9:**
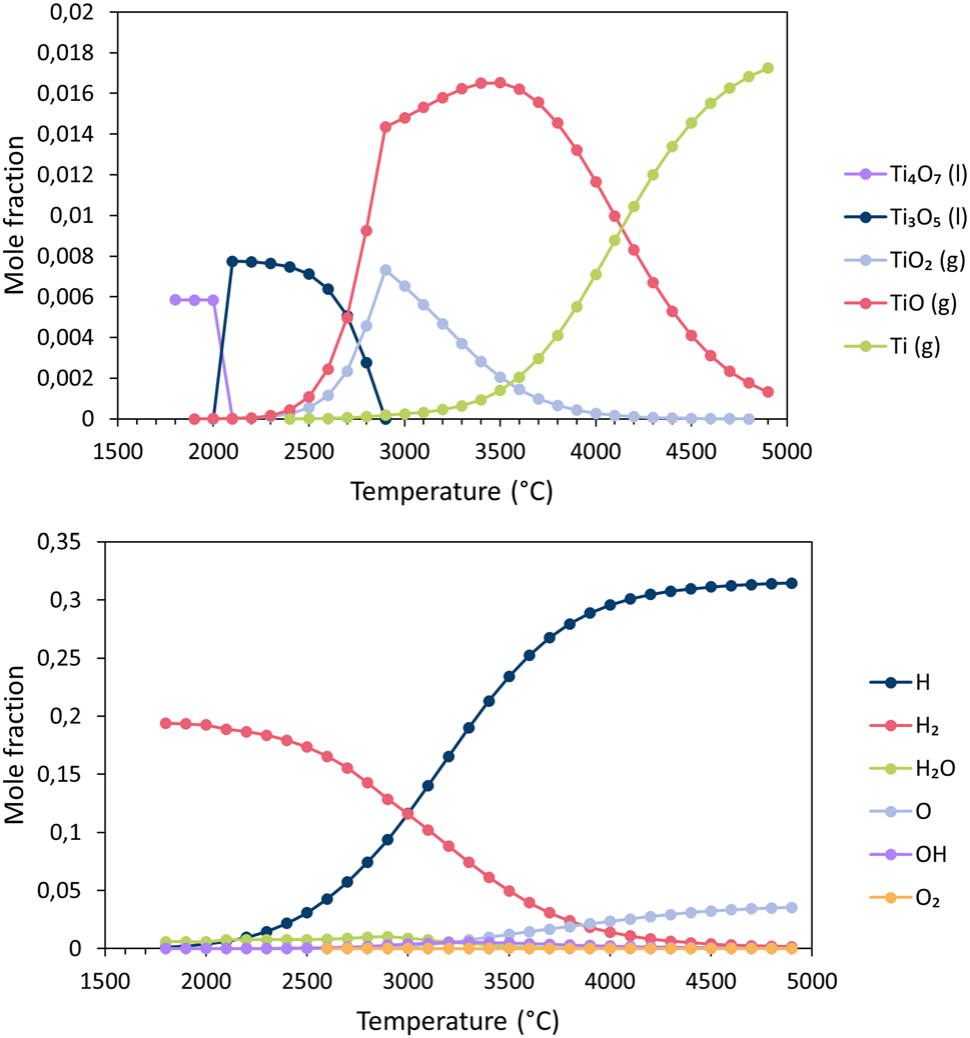
Melting, vaporization, and dissociation of TiO_2_ in Ar–H_2_ atmosphere (case 2) at chemical equilibrium; top: Ti-containing species; bottom: H-containing species and O; balance Ar, *p* = 0.1 MPa.

The addition of H_2_ into the plasma, as shown in Fig. 9, shifts the onset temperatures of all dissociation and decomposition events to significantly lower values compared those shown in Fig. 8 for case 1. Surprisingly, only a very small amount of the H_2_ in the plasma must be consumed for this reduction to occur; the excess gas simply dissociates to atomic H. In the Ar-H_2_ plasmas, liquid-phase stoichiometric TiO_2_ does not exist, despite being present in the Ar-only case 1. This can be deemed another influence of the addition of hydrogen in the plasma gas mixture. Additionally, the lack of stoichiometric TiO_2_ liquid phase in Ar–H_2_ plasmas can serve as a direct explanation toward why the work of Lee et al. found that the Ar-only spraying parameters were most thermoelectrically-optimal—as they yielded some, yet minimal, TiO_2_ reduction and the most rutile phase retention^10,14^.

The onset of Ti vapor formation in this new Ar–H_2_ case 2 takes place at approx. 2900 °C. The nature of the chemical equilibria curves was found to be quite similar between case 2 and case 3 (where only the argon flow rate was increased). For the increased hydrogen case 4, these curves were found to be slightly shifted to even lower temperatures compared to cases 2 and 3, which is consistent with the hypothesis that additional hydrogen in the plasma reduces these onset temperatures.

In addition, it is known from Fig. 2 that the introduction of hydrogen in the plasma dramatically increases the particle temperatures at any given standoff distance. Therefore, one could consider that the hydrogen effect on TiO_2_ is twofold: not only reducing the necessary temperature for the onset of dissociation, but also drastically increasing particle temperatures such that they approach or exceed this notional threshold temperature.

From these OES results, it is clear that adding hydrogen in the APS process has an unavoidable reducing effect on the TiO_2_ feedstock (and the sub-stoichiometric Ti oxides that first form). It was also found that only small amounts of hydrogen are required to reveal this phenomenon for all the spraying conditions in this work. In fact, to reduce 5 g min^−1^ of the TiO_2_ feedstock completely to TiO, only ~ 0.35 slpm H_2_ would be required, and again the same amount of H_2_ would be needed to reduce this TiO to Ti. Therefore, it is likely to expect a more reduced TiO_2−x_ coating for Ar–H_2_ spraying conditions, which further corroborates what is seen in Fig. 3b, as coatings with heterogeneous through-thickness phase distributions are formed.

### Calculated Ti concentrations

From the OES measurements, relative concentrations of Ti atoms in the plasma jet stream can be evaluated based on the hydrogen content in the flame using the equations laid out in "Species concentrations in the plasma" section. The highest Ti I-concentrations were found at 65Ar + 11H_2_ (case 4), followed by 65Ar + 6H_2_ (case 3) and 47.5Ar + 6H_2_ (case 2). In this way, the vaporization and dissociation of TiO_2_ could be quantified on a relative scale.

**Figure 10:**
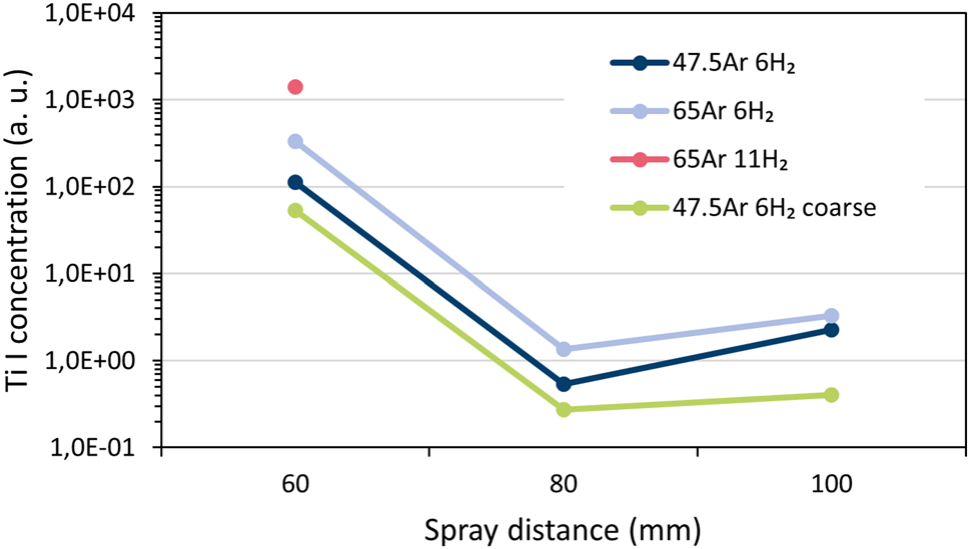
Concentrations of Ti atoms determined by OES for three spray distances and four experimental cases.

In addition to studying the effect of the plasma gas compositions, OES measurements were taken at multiple torch standoff distances. In doing so, the nature of the inflight reduction of TiO_2_ as a function of particle flight time/distance can be ascertained. At the shortest standoff distances of 60 mm, the Ti I-concentrations were observed to be highest, before sharply dropping to lower levels at standoff distances 80 and 100 mm, see Fig. 10. This sharp decrease from 60 to 80 mm could be due to the recombination of Ti to TiO and TiO_2_ in the atmosphere (and, to a smaller extent, due to the divergence and the pressure distribution of the jet). By the same logic, the slight increase in Ti concentration seen from 80 to 100 mm could be due to the recombination of some Ti II-ions and electrons to neutral Ti I atoms. This ionic recombination can be another source of energy that contributes to why the plasma temperatures remain in the same range across all standoff distances, as shown in Fig. 4. The release of both dissociation and ionization energy from the TiO_2−x_ material could have contributed to keep the plasma temperatures at 80 and 100 mm high (cp. "Calculated plasma temperatures" section, Fig. 4).

The ranking of the Ti I-concentrations is seen to be inverse to the plasma temperatures. For instance, with the 65Ar–11H_2_ case 4, the lowest plasma temperatures yet conversely the highest Ti concentrations were observed. Obviously, one can consider the Ti concentrations should increase as more thermal energy is transferred from the plasma to the powder. This increase in thermal energy transfer logically stems from heat transfer in the APS process being a matter of both plasma enthalpy and thermal conductivity. When either plasma enthalpy or plasma thermal conductivity increase, the Ti concentrations should subsequently increase. In this study, as argon flow rates increase (from case 2 → case 3), as described earlier, the mass specific enthalpy of the plasma increases; furthermore, as hydrogen flow rates increase (from case 3 → case 4), the thermal conductivity of the plasma increases. Hence, it was experimentally observed that stepping through the process in this iterative fashion (from case 1 $\to$ case 4) yields progressively higher Ti vapor concentrations. Lastly, it is interesting to note that at the 60 mm standoff distance, there is almost an order of magnitude difference in Ti I-concentration for the 65Ar + 11 H_2_ (case 4) plasma compared to the 47.5Ar + 6 H_2_ (case 2). This is surprising, considering it was previously determined that only a small amount of hydrogen gas (in slpm) would be required to reduce TiO_2_ to TiO and subsequently Ti in the plasma. Yet, clearly, it seems plasma conditions with even higher hydrogen contents (i.e., 11 slpm, case 4), can produce consistently higher Ti concentrations than other cases. This is also consistent with Lee et al.’s work, which demonstrated that spraying conditions with excessively high hydrogen flow rates yield thermoelectrically-poor TiO_2−x_^10,13,14^.

The lowest Ti I-concentrations among all the datasets examined were found for the coarser powder. The reason is obviously less evaporation can take place given the relatively smaller area-to-volume ratio of the coarse powder.

## Conclusions

This study has strived to demonstrate several key points that will enable future researchers to develop new process diagnostic methodologies for the APS processing of complex oxides. These points are as follows.

First, it was found that despite the challenges of using Ar I optical emission lines at atmospheric pressures (due to concurrent continuum emission of recombination and bremsstralung and due to considerable peak broadening), it is indeed possible to carry out semi-quantitative Optical Emission Spectroscopy measurements in the APS process. It was found that by using TiO_2_ feedstock material (under appropriate conditions, i.e., wherein the Ti particles are at a high enough temperature to undergo dissociation and vaporization), the Ti I emission lines can be used to fit Boltzmann plots and calculate the plasma temperatures to a very high degree of accuracy. From here, the concentrations of evaporated species and chemical equilibria can then be estimated by using these plasma temperatures as input parameters. These findings have implications that TiO_2_ (or Ti) powder could be used as a so-called ‘marker’ material in APS for studying the reduction or dissociation of other key materials in the thermal spray community.

Additionally, this study determined the sequence of events that lead to inflight reduction of TiO_2_ in the APS process. It was found that, generally speaking, the TiO_2_ feedstock should become liquid, then undergoes dissociation into Ti_n_O_2n−1_ liquid phases, followed by subsequent vaporization and dissociation into TiO + O and Ti + O. At longer spray distances, recombination back into TiO and TiO_2_ can occur. The presence of hydrogen in the plasma flame drastically reduces the onset temperatures of these events, which in turn can contribute to the formation of coatings which are heterogenously phase-segreated after deposition.

## Experimental methods

### Atmospheric plasma spraying

Spray experiments were carried out at the Jülich Thermal Spray Center (JTSC)^31^ on a Multicoat atmospheric plasma spray (APS) system [Oerlikon Metco, Wohlen, Switzerland] using a F4-MB torch with an Ø8 mm nozzle. The process parameters are summarized in Table 1. Case 1 from the Table is taken directly from past works of Lee et al.^14^; the additional conditions are analogous steps in the processing space for the F4-MB spray torch to iteratively determine the effect of hydrogen content in the plasma gas mixture. The torch standoff distance varied between 60 and 150 mm. For coating depositions, a ladder meander pattern was utilized wherein the torch travel velocity (relative to the stationary substrate) was 250 mm s^−1^, and the raster step was 2 mm. The average temperature and velocity of individual droplets within the spray plume were measured using commercial hardware [DPV Millenium Edition, Tecnar Automation Ltd., QC CA]. Briefly, the sensor determines the centermost region of the spray plume at any given condition, then using two-color pyrometry and a two-slit mask, the temperature of molten droplets of material as well as their absolute velocity (in m s^−1^) can be determined^32^. From the data, a distribution of temperatures and velocities is acquired. Shown in this paper are the average values of temperature and velocity for the given spraying conditions. As the plasma parameters (i.e., input gas flows) change, the required carrier gas (C/G) necessary to entrain the particles into the centermost hot core of the plasma also changes. Using methods described elsewhere, particle injection optimization was carried out to ensure optimal C/G flow rates^33^.

The feedstocks used here were commercially-available fused and crushed powders: Amperit 782.008 (−20/+5 µm) and Amperit 782.001 (−45/+22 µm), both from Höganäs, formerly H.C. Starck [Höganas Germany GmbH, Goslar, Germany]. In the following, the latter is referred to as ‘coarse’ powder. Because there was limited quantity of the fine powder during the experiments (and is no longer available in the supplier’s assortment), it was pertinent to also study concurrently the coarse powder and evaluate what (if any) size effects may exist. The powder mass feed rate for both feedstocks was kept constant at 5 g min^−1^. The characteristic particle diameters determined by laser diffraction [Horiba LA 950 V2, Retsch Technology, Germany] are given in Table 2.

**Table 2:** Characteristic diameters of TiO_2_ powders.

	Amperit 782.008	Amperit 782.001 (coarse)
d_10_ (µm)	7.9	18.8
d_50_ (µm)	12.9	31.7
d_90_ (µm)	19.3	47.1

### Coating Characterization

Several coatings were deposited as part of this study to assess the as-deposited microstructure due to changes in the spray process. For this, coatings were sprayed onto VA Steel substrates (25 × 25 × 3 mm). Before spraying, the substrates were pre-cleaned by ultrasonic cleaning in ethanol, then grit blasted using F36 grit at a pressure of 4 bar. This allows for the surface of the specimen to be nominally around R_a_ 5 µm prior to deposition—which allows for the necessary mechanical interlocking for thermally-sprayed coating adhesion.

After deposition, coatings were then sectioned using a diamond saw [Accutom, Struers GmbH, Willich, Germany], then embedded in a 2-part epoxy [EpoFix, Struers GmbH, Willich, Germany] by vacuum-impregnation. Once cured, the epoxy-mounted specimen was polished using standard metallographic methods, with a final colloidal silica (~ 500 nm agglomerate size) finishing step. Then the samples were imaged using a LaB_6_ cathode SEM [Zeiss EVO15, Carl Zeiss Inc., United Kingdom] in the backscatter mode as well as a Raman Optical Microscope [Renishaw InVia Qontor, Renishaw Inc., United Kingdom]. Raman spectroscopy measurements were carried out on the cross-sections of one of these samples to determine the phase heterogeneity. For this, a 532 nm laser with 5 mW was used as the excitation source.

### Optical emission spectroscopy

#### Plasma temperatures

The spectrometer applied for optical emission spectroscopy (OES) was the ARYELLE 200 model [Laser Technik Berlin (LTB), Berlin, Germany], scanning a wavelength range between 381 and 786 nm. The OES measurement position was fixed to be perpendicular to the torch axis and the spray torch was manipulated by a six-axis robot to achieve measurements at multiple standoff distances. The plasma radiation was collected and transferred through an achromatic lens and an optical fiber to the 50 µm entrance slit of the spectrometer, and then detected by a 1024 × 1024 CCD array. The system is equipped with an Echelle grating, and the spectral resolution capability is 22,000 (17.3–35.7 pm). Calibration was carried out using a spectral mercury lamp.

The plasma temperatures were determined by intensity analyses of specific emission lines applying the Boltzmann plot method. The integrated intensity of a spectral line emitted by the plasma due to the transition from an excited energy state *k* to a lower state *i* is expressed as follows^34^,:

1 $$I_{ki} = \frac{L}{4\pi }A_{ki} n_{tot} \frac{{g_{k} }}{Z\left( T \right)}\frac{h c}{\lambda } \exp \left( {\frac{{ - E_{k} }}{{k_{B} T_{exc} }}} \right)$$

where *L* is the emission source depth, *A*_*ki*_ is the transition probability, *n*_*tot*_ is the total density of a plasma species, *g*_*k*_ is the statistical weight (degeneracy) of the excited level *k, Z(T)* is the partition function (sum over states), *h* is the Planck constant 6.626⋅10^−34^ J s, *c* is the velocity of light 299,792,458 m s^−1^,* λ*_*ki*_ is the wavelength of the emission, *E*_*k*_ is the energy level of the excited state *k*, *k*_*B*_ is the Boltzmann constant 8.617‧10^−5^ eV K^−1^, and *T*_*exc*_ is the excitation temperature.

Rearranging Eq. (1) to make it take the form of a straight line $y=m {E}_{k}+q$ with the slope *m* and the intercept *q* gives the atomic state distribution function (ASDF):

2 $$ASDF = \ln \left( {\frac{{I_{ki} \lambda_{ki} }}{{A_{ki} g_{k} }}} \right)$$

In Boltzmann plots, *y* = ASDF is plotted against *x* = *E*_*k*_ for a set of emission lines of the same species and ionization state^35^. These data points are fitted by a straight line, and with its slope *m*, the excitation temperature *T*_*exc*_ is calculated by:

3 $$T_{exc} = \frac{ - 1}{{m k_{B} }}$$

The uncertainty of *T*_*exc*_ can be estimated on the basis of the standard error of the slope. More details on this technique can be found in a previous publication^19^. The emission line data were taken from NIST Atomic Spectra Database line tables^36^; moreover, some nodes of the partition functions *Z*(*T*) were calculated based on the atomic energy level tables ibid. and then fitted by quadratic regression functions to obtain arbitrary intermediate values (*T* must be inserted in K):

$$Z\left( T \right) = 8 \cdot 10^{ - 7} T^{2} - 0.0024\,T + 20.47\quad {\text{for}}\;{\text{neutral}}\;{\text{Ti}}\;{\text{I}}\;{\text{and}}$$

$$Z\left( T \right) = - 2 \cdot 10^{ - 8} T^{2} + 0.0058\,T + 26.72\quad {\text{for}}\;{\text{singly}}\;{\text{ionized}}\;{\text{Ti}}\;{\text{II}}.$$

In this work, the line integral intensities *I*_*ki*_ were obtained by fitting the measured emission line data points by Voigt functions^37^. Voigt profiles are a convolution of a Lorentz and a Gauss profile taking into account different line broadening mechanisms^38^. The line intensities were obtained by taking the integral of the Voigt profiles over the wavelength.

Boltzmann plots require local thermal equilibrium (LTE) to be assumed. Under LTE conditions, the resulting excitation temperature *T*_*exc*_ can be equated to electron temperature *T*_*e*_ and also to temperature of the heavy species *T*_*h*_ (atoms and ions)^34^, i.e.,:

4 $$T_{exc} = T_{e} = T_{h} \equiv T$$

Under atmospheric pressures (as in this work), LTE can be regarded as given, in any case, in the center of the plasma jet.

In contrast to atmospheric conditions, the work referenced above^19^ was performed under low pressure/vacuum conditions at 150 Pa. A set of fifteen Ar I lines was used for the Boltzmann plots considering only transitions with high energy levels of the excited states *E*_*k*_ (transitions from 6 s, 6d, 5d, and 4d, respectively, to 4p) to comply with LTE. In the present work however, the detectability of all these Ar I lines was affected by the superimposed strong continuum emission (recombination radiation and bremsstrahlung). These alternative emission sources were on a much higher level compared to low pressure plasma spraying (LPPS) process due to the atmospheric pressure in this work’s case. Drawing Boltzmann plots with an alternative set with eight Ar I lines of transitions from low energy levels *E*_*k*_ (transitions 4p to 4 s) at wavelengths above 696 nm was attempted in this work. However, this proved also to be difficult, as several molecular bands obviously originating from air entrained into the plasma jet overlapped with these alternative Ar I lines, in particular at higher plasma enthalpies (i.e., cases 2–4 in Table 1). Moreover, the quality of the linear fits in the Boltzmann plots was poor. This is because the energy levels of the excited states *E*_*k*_ were closely packed. Consequently, the resulting calculated temperatures showed high uncertainties—larger than 1000 K. The corresponding coefficients of determination were generally below 0.5 (only in one case about 0.6). It is for these reasons that the application of OES in atmospheric pressure conditions has proven challenging in the past.

**Figure 11:**
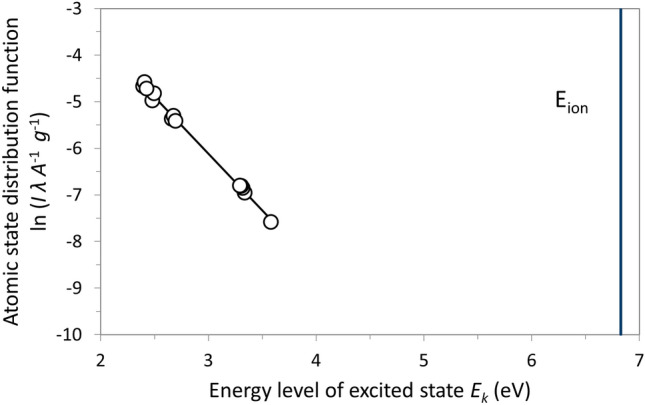
Example of a Boltzmann plot for case 2 (see Table 1) using Ti I emission lines, spray distance 100 mm; the slope of the straight line corresponds to a temperature of 4773 K, the uncertainty resulting from the standard deviation of the slope is ± 81 K, the coefficient of determination of the linear regression is 0.997.

However, recent published work has shown that lines of neutral titanium were found to be much more suitable in these conditions^39^. In this study, thirteen Ti I lines in the wavelength range between 453 and 521 nm were selected for Boltzmann plots. Most of these lines were also used in the work of Menneveux et al.^17^. Of course, these lines are only present when there is simultaneous powder injection while the plasma torch is operational—wherein TiO_2_ is vaporized. Using these Ti I lines, the linear fits in the Boltzmann plots yielded coefficients of determination better than 0.99, confirming that the excitation energy of the Ti atoms followed a Boltzmann distribution. The uncertainties of the resulting temperatures were correspondingly small. See Fig. 11 for an example. Only in the case 1 (see Table 1) where only argon was used as a plasma gas (thereby yielding low plasma enthalpy), these Ti I lines were too minute to be evaluated. This limiting scenario was also found for the other experimental cases 2, 3, and 4 at the longest standoff distance of 150 mm.

#### Species concentrations in the plasma

If the radiating transitions are Boltzmann-distributed, the intensity of a spectral line shows a duplex dependency on the temperature and the concentration of the radiating species, cp. Eq. (1). It can be rearranged so that the total density of a plasma species is proportional to

5 $$n_{tot} \propto \frac{{I_{ki} Z\left( T \right) \lambda_{ki} }}{{g_{k} A_{ki} }} \exp \left( {{{E_{k} } \mathord{\left/ {\vphantom {{E_{k} } {k_{B} T}}} \right. \kern-0pt} {k_{B} T}}} \right)$$

The temperature *T* enters into the partition function *Z(T)* and into the exponential Boltzmann factor. This temperature can be determined by means of Boltzmann plots as mentioned above. The concentration of specific plasma constituents *n*_*tot*_ can be determined on the basis of a single emission line, or alternatively several lines can be used and the corresponding calculated concentrations then averaged. More details of the method are given in a previous publication^18^. It is calibration-free and yields concentrations *n*_*tot*_ in arbitrary units, which are quantitatively comparable with each other and thus self-consistent. The required input emission line data were taken again from the NIST Atomic Spectra Database line tables^36^ and the partition functions *Z*(*T*) were calculated as mentioned in "Plasma temperatures" section.

Quantitative analyses require emission lines from a plasma which is free from self-absorption and optically thin. To minimize self-absorption effects, two criteria were proposed^40^: (1) transitions should be avoided with a lower energy level *E*_*i*_ less than 6000 cm^−1^ (approx. 0.74 eV) or (2) with a transition probability *A*_*ki*_ below 2⋅10^6^ s^−1^. The five most powerful Ti I-lines at 453.324 nm, 498.1731 nm, 499.1067 nm, 499.9502 nm, and 500.7206 nm are all beyond those two thresholds. They are all associated to 4p → 4 s transitions.

**Figure 12:**
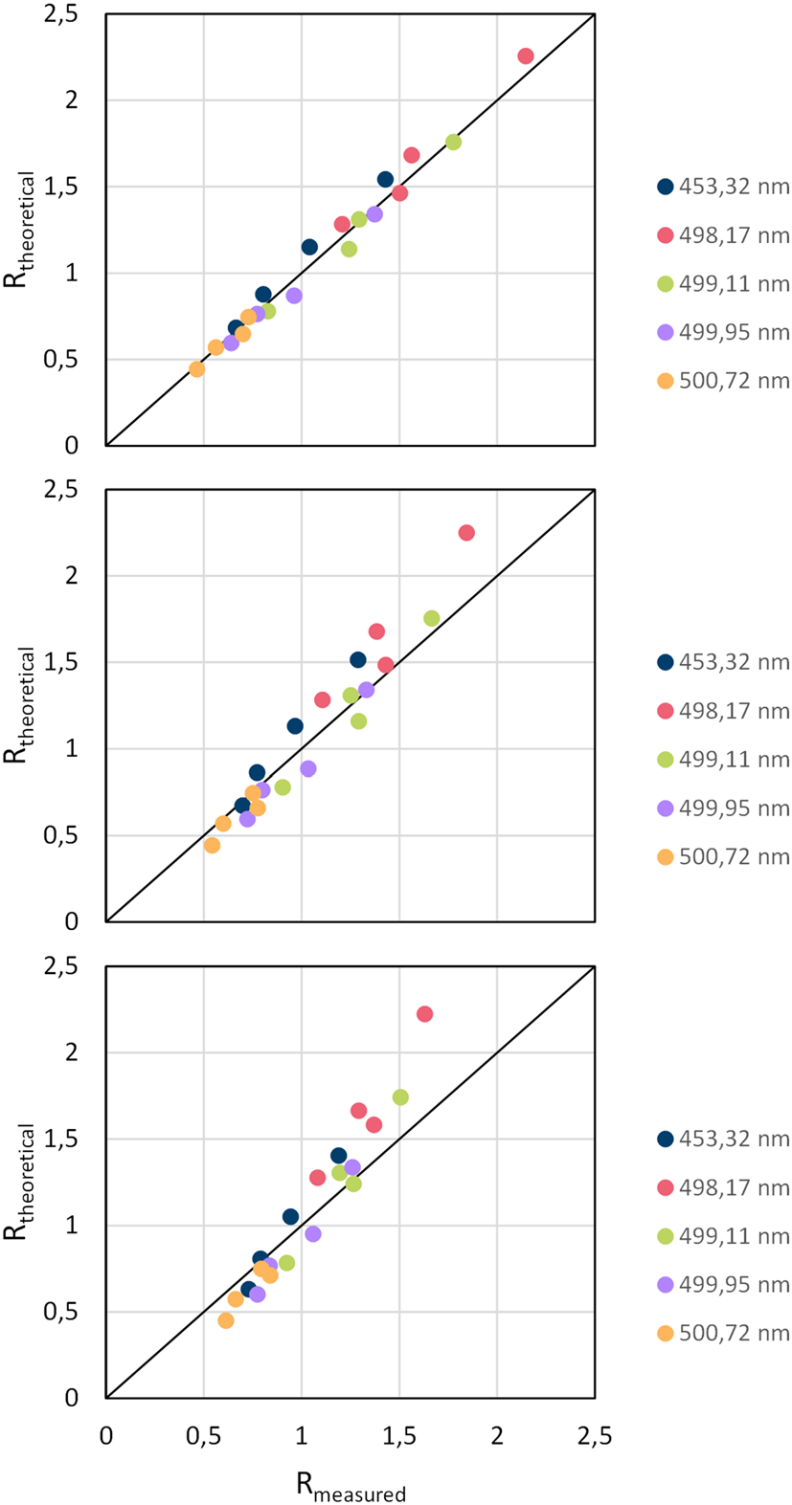
Plots of the calculated theoretical intensity ratio against the measured intensity ratio for the experimental cases 2, 3, and 4 at a spray distance of 100 mm; the 1:1-straight line is also drawn.

The condition of an optically-thin plasma can be assessed by calculating the theoretical intensity ratio ${R}_{theoretical}={I}_{ki}^{(a)}/{I}_{ki}^{(b)}$ of two lines (*a*) and (*b*) of the same element and the same charge state using Eq. (1). However, reliable temperature data is needed for this. This theoretical intensity ratio is then compared with the ratio ${R}_{measured}$ of two measured line intensities^40,41^. Ideally, both ratios would match. The theoretical ratios were calculated for all possible combinations of the five mentioned Ti I-lines and plotted against the corresponding measured ratios. If the ratios are equal, data points are located on the 1:1-straight line. Figure 12 shows three plots for the experimental cases 2, 3, and 4 (see Table 1) and a spray distance of 100 mm. Generally, the theoretical and measured intensity ratios agree well. However, with increasing plasma power, small deviations occur—indicating a slight tendency toward optical thickness. The following evaluation of the concentrations was done based only on the one Ti I-line at 499.1067 nm—which from this initial comparison in Fig. 12 showed the overall best agreement of theoretical and experimental intensity ratios. Nevertheless, the evaluation of a preliminary set of experiments showed that all the mentioned five Ti I-lines yielded very similar Ti I concentrations (not shown here).

#### Electron density and characteristic plasma parameters

The Electron density *n*_e_ can be calculated applying the Saha–Eggert equation^42^:

6 $$\frac{{n}_{e} {n}_{1}}{{n}_{0}}=\frac{2 {Z}_{1}(T)}{{Z}_{0}(T)}\frac{2}{{\lambda }_{th}^{3}} exp\left(-\frac{{E}_{1}}{{k}_{B} T}\right)$$

where *n* is the particle density, *E*_1_ the ionization energy, and subscripts 0 and 1 denote the neutral and singly ionized state, respectively; the thermal de Broglie wavelength *λ*_th_ is:

7 $${\lambda }_{th}\equiv \sqrt{\frac{{h}^{2}}{2\pi {m}_{e} {k}_{B} T}}$$

where *m*_e_ = 9.1⋅10^−31^ kg is the mass of an electron. The ratio *n*_1_/*n*_0_ can be calculated applying Eq. (5) for emission lines of singly ionized and neutral Ti II and Ti I, respectively.

The Debye screening length *λ*_D_ is a characteristic plasma dimension and as such a measure of the range of electrostatic interactions in the plasma:

8 $${\lambda }_{D}=\sqrt{\frac{{\varepsilon }_{0} {k}_{B} {T}_{e}}{{n}_{e} {e}^{2}}}$$

where *ε*_0_ = 8.86⋅10^−12^ A s V^−1^ m^−1^ is the vacuum permittivity and *e* = 1.6⋅10^−19^ A s is the charge of an electron. *λ*_D_ results from the condition that in this distance from a positive ion its Coulomb field is shielded by electrons since the potential of the ion’s charge decreases to the eth part^26^ (e is Euler's number). Such an accumulation of negative charges in the vicinity of a positive ion represents a net-negative space charge, i.e., a deviation from charge neutrality occurs over the dimension of this electron cloud, which is known as a Debye sphere^34^. Within, the condition of quasineutrality is disturbed. Thus, the overall electrical neutrality of a plasma and hence collective interaction phenomena as they are typical for a plasma apply only for sufficiently large volumes extending well beyond the Debye sphere.

The average number of electrons in a Debye sphere with radius *λ*_D_ is the Debye number:

9 $${N}_{D}={n}_{e}\frac{4}{3}\pi {\lambda }_{D}^{3}=\frac{4\pi {\left({\varepsilon }_{0} {T}_{e}{k}_{B}\right)}^{3/2}}{3 {n}_{e}^{1/2} {e}^{3}}\equiv\Lambda$$

*N*_D_ is defined as plasma parameter Λ (some authors use the inverse definition). A true plasma with continuity conditions can only exist if Λ ≫ 1.

The Landau length *λ*_L_ is the smallest characteristic scale in a plasma^43^. At this distance, potential and kinetic energy balance each other in an electron–ion encounter^34^ (some authors use ‘4’ instead of ‘6’, as they do not average the particle’s kinetic energy):

10 $${\lambda }_{L}=\frac{{e}^{2}}{6\pi {\varepsilon }_{0} {k}_{B} {T}_{e}}$$

To prevent an ion and electron from recombination, their average interparticle distance (Wigner–Seitz radius) must be larger than the Landau length *λ*_L_:

11 $$\overline{x }=\sqrt[3]{\frac{3}{4\pi {n}_{e}}}>{\lambda }_{L}$$

On the other hand, since the distance of the electrostatic interaction of plasma particles is the Debye length *λ*_D_, only the relation $\overline{x }>{\lambda }_{D}$ means that there is no cooperative interaction between particles, i.e. there is no plasma^26^. In this work, the electron density, the characteristic plasma lengths and the average particle distance were applied as important key figures to assess the state of the plasma in the investigated experimental cases.

## Data Availability

The datasets generated during and/or analyzed during the current study are available from the corresponding author on reasonable request.
